# Supplementary material for: uPA‐PAI‐1 heteromerization promotes breast cancer progression by attracting tumorigenic neutrophils
Source: EMBO Mol Med. 2021 May 16;13(6):e13110. doi: 10.15252/emmm.202013110 (PMC8185543; doi:10.15252/emmm.202013110)
Supplement: Supplementary file 1 — Appendix [file EMMM-13-e13110-s003.pdf]

APPENDIX

Appendix tables S1, S2, and S3

Appendix figures S1, S2, S3, and S4

Appendix tables

specimen number	histology	grade	PAI-1 protein [ng/ml]	uPA protein [ng/ml]	extravascular neutrophils [n / mm^2]	intravascular neutrophils [n / mm^2]
1	mixed invasive lobular/ductal carcinoma	G1	10.83	6.64	782.68	545.50
2	invasive ductal carcinoma	G1	14.49	5.67	1,423.29	1,501.63
3	invasive ductal carcinoma	G1	28.43	6.66	1,657.56	4,490.48
4	mixed invasive lobular/ductal carcinoma	G1	12.23	1.76	1,164.05	2,993.28
5	mixed invasive lobular/ductal carcinoma	G1	46.55	7.97	3,450.43	4,540.04
6	mixed invasive lobular/ductal carcinoma	G2	9.40	0.92	2,875.82	3,229.48
7	invasive ductal carcinoma	G2	15.64	3.32	1,028.08	639.07
8	invasive ductal carcinoma	G2	9.40	1.48	2,684.73	1,534.13
9	invasive lobular carcinoma	G2	27.75	3.68	625.64	2,027.54
10	invasive lobular carcinoma	G2	6,13	0,54	2,174.57	1,199.76
11	invasive lobular carcinoma	G2	16.98	1.73	4,114.31	16,228.70
12	invasive ductal carcinoma	G2	37.61	2.89	3,440.92	4,697.66
13	invasive ductal carcinoma	G2	25.68	1.55	274.74	595.28
14	invasive lobular carcinoma	G2	32.17	5.63	1,723.48	3,770.12
15	invasive ductal carcinoma	G2	35.01	5.36	3,133.51	3,240.09
16	invasive ductal carcinoma	G2	16.05	1.65	708.60	2,000.75
17	invasive ductal carcinoma	G2	12.00	2.68	4,279.50	4,054.26
18	mixed invasive lobular/ductal carcinoma	G2	20.54	1.86	1,094.44	2,736.09
19	invasive lobular carcinoma	G2	14.22	1.78	1,528.97	1,868.74
20	invasive micropapillary carcinoma	G2	10.81	3.32	989.33	2,398.78
21	mixed invasive lobular/ductal carcinoma	G2	23.68	3.76	412.04	1,381.53
22	invasive ductal carcinoma	G2	29.91	4.57	1,689.91	3,869.00
23	invasive lobular carcinoma	G2	20.35	2.30	355.83	1,392.37
24	invasive lobular carcinoma	G2	40.55	0.52	6,283.55	11,753.17
25	invasive ductal carcinoma	G2	18.52	2.99	978.07	2,941.62
26	invasive ductal carcinoma	G2	10.11	1.62	2,366.36	5,733.88
27	invasive ductal carcinoma	G2	13.95	6.85	668.17	2,111.41
28	invasive ductal carcinoma	G2	20.77	5.23	1,038.76	2,095.13
29	invasive lobular carcinoma	G2	29.59	2.92	2,400.83	3,906.34
30	invasive ductal carcinoma	G2	21.42	6.68	2,548.16	3,136.71
31	invasive ductal carcinoma	G2	21.03	5.28	1,165.98	4,387.78
32	invasive ductal carcinoma	G2	23.92	7.24	2,695.33	5,003.99
33	invasive ductal carcinoma	G2	48.69	7.56	583.23	1,621.37

34	invasive lobular carcinoma	G2	13.44	2.82	3,428.36	3,795.68
35	invasive ductal carcinoma	G3	16.76	9.91	890.42	1,307.80
36	invasive lobular carcinoma	G3	23.88	6.03	92.15	506.81
37	invasive ductal carcinoma	G3	15.01	6.17	7,298.70	15,977.51
38	invasive ductal carcinoma	G3	13.10	2.23	476.44	1,577.61
39	invasive ductal carcinoma	G3	10.01	1.29	1,022.30	3,553.12
40	invasive lobular carcinoma	G3	4.90	1.65	741.76	1,335.16
41	invasive ductal carcinoma	G3	19.97	3.59	3,779.61	7,447.35
42	invasive lobular carcinoma	G3	41.61	6.68	30,610.96	93,101.13
43	invasive ductal carcinoma	G3	194.82	9.35	5,311.23	6,030.04
44	invasive ductal carcinoma	G3	10.01	1.29	570.91	1,534.33

Appendix table S1. Characteristics of human breast cancer samples. Histopathological characteristics including histological subype and grade as well as uPA/PAI-1 protein content and neutrophil accumulation in the analyzed breast cancer samples are shown.

antibody	systemic neutrophil count [x 10^3 μl^{-1}]
isotype control	4.09 ± 0.2
anti-Ly-6G mAb (clone 1A8)	0.04 ± 0.1#

antibody	systemic cMO count [x 10^3 μl^{-1}]
isotype control	0.41 ± 0.04
anti-CCR2 mAb (MC-21)	0.03 ± 0.01#

Appendix table S2. Depletion of neutrophils and cMOs. Systemic counts of neutrophils and cMOs in the peripheral blood of 4T1 tumor-bearing WT mice receiving neutrophil-depleting (anti-Ly-6G), cMO-depleting (anti-CCR2), or isotype control antibodies. Quantitative data are shown (mean±SEM for n=7; #p<0.05 vs. isotype control).

figure	n	p value	statistical test
1a (left, neutrophils)	5	< 0.0001	One-way ANOVA
1a (left, cMOs)	5	< 0.0001	One-way ANOVA
1a (left, ncMOs)	5	0.0217	One-way ANOVA
1a (right, B cells)	5	0.7697	t test
1a (right, CD4+ cells)	5	0.6088	t test
1a (right, CD8+ cells)	5	0.7311	t test
1b (neutrophils)	4	0.002	One-way ANOVA
1b (cMOs)	4	0.0012	One-way ANOVA
1c (neutrophil rolling)	4	0.9983	One-way ANOVA
1c (neutrophil adherence)	4	< 0.0001	One-way ANOVA
1c (neutrophil transmigration)	4	< 0.0001	One-way ANOVA
1c (cMO rolling)	4	0.1212	One-way ANOVA
1c (cMO adherence)	4	< 0.0001	One-way ANOVA
1c (cMO transmigration)	4	< 0.0001	One-way ANOVA
2b (ICAM-1)	4	0.0436	t test
2b (VCAM-1)	4	0.0428	t test
2c	4	< 0.0001	One-way ANOVA
2d (ICAM-1)	4	< 0.0001	One-way ANOVA
2d (VCAM-1)	4	< 0.0001	One-way ANOVA
2d (uPA)	4	0.6479	t test
2d (PAI-1)	4	0.0108	t test
2e (ICAM-1)	4	0.0219	One-way ANOVA
2e (VCAM-1)	4	0.9798	One-way ANOVA
2f (kim127)	6	0.0499	One-way ANOVA
2f (mAB24)	6	0.0096	One-way ANOVA
2g	30	< 0.0001	One-way ANOVA
3c (MMP-9)	4-6	0.8857	t test
3c (VEGF)	4-6	0.4688	t test
3c (NE)	6	0.0174	t test
3d (left)	6	0.6109	One-way ANOVA
3d (mid)	3	0.0125	t test
3d (right)	3	0.0088	t test
4a (0)	3	< 0.0001	One-way ANOVA
4a (0.1)	3	0.0005	One-way ANOVA
4a (1)	3	< 0.0001	One-way ANOVA
4a (10)	3	< 0.0001	One-way ANOVA
4b (neutrophil rolling)	6	0.9345	One-way ANOVA
4b (neutrophil adherence)	6	< 0.0001	One-way ANOVA
4b (neutrophil transmigration)	6	< 0,0001	One-way ANOVA
4b (cMO rolling)	6	0.213	One-way ANOVA
4b (cMO adherence)	6	< 0.0001	One-way ANOVA
4b (cMO transmigration)	6	< 0.0001	One-way ANOVA
4d	6	0.0091	One-way ANOVA
4e (rolling)	4-6	0.1948	t test
4e (adherence)	4-6	0.0105	t test
4f (lung neutrophils)	4	0.0413	t test
4f (lung tumor cells)	4	0.0489	t test
4f (brain neutrophils)	4	0.0012	t test
4f (brain tumor cells)	4	0.0456	t test
EV 1b (IL-6)	5	0.0029	t test
EV 1b (TNF)	5	< 0.0001	t test
EV 1b (CXCL2)	5	0.0059	t test
EV1b (CCL4)	5	< 0.0001	t test

EV2a (left, neutrophils)	4	0.0274	One-way ANOVA
EV2a (left, cMOs)	4	0.0015	One-way ANOVA
EV2a (right, neutrophils)	4	0.0002	One-way ANOVA
EV2a (right, cMOs)	4	< 0.0001	One-way ANOVA
EV2b (left, neutrophils)	4	0.0256	One-way ANOVA
EV2b (left, cMOs)	4	0.0006	One-way ANOVA
EV2b (right, neutrophils)	4	0.066	One-way ANOVA
EV2b (right, cMOs)	4	0.0003	One-way ANOVA
EV2c (neutrophils)	4	0.012	One-way ANOVA
EV2c (cMOs)	4	0.0081	One-way ANOVA
EV3a (macrophages)	4	0.0215	t test
EV3a (neutrophils)	4	0.0011	t test
EV3b	4	0.0440	One-way ANOVA
EV3c (left)	4	< 0.0001	One-way ANOVA
EV3c (right)	4	0.002	One-way ANOVA
Appendix Tab 2 (Ly-6G)	7	< 0.0001	t test
Appendix Tab 2 (CCR2)	7	< 0.0001	t test
Appendix Fig S1a (neutrophil rolling)	4	0.6966	t test
Appendix Fig S1a (neutrophil adherence)	4	0.6536	t test
Appendix Fig S1a (neutrophil transmigration)	4	0.7970	t test
Appendix Fig S1a (cMO rolling)	4	0.4831	t test
Appendix Fig S1a (cMO adherence)	4	0.5546	t test
Appendix Fig S1a (cMO transmigration)	4	0.7420	t test
Appendix Fig S1b (ICAM-1)	6	0.0014	One-way ANOVA
Appendix Fig S1b (VCAM-1)	6	0.0002	One-way ANOVA
Appendix Fig S2a	4	0.0002	t test
Appendix Fig S2b (left, ICAM-1)	6	0.0002	t test
Appendix Fig S2b (left, VCAM-1)	6	< 0.0001	t test
Appendix Fig S2b (right, ICAM-1)	6	< 0.0001	t test
Appendix Fig S2b (right, VCAM-1)	6	< 0.0001	t test
Appendix Fig S2d (left)	4	0.8803	t test
Appendix Fig S2d (right)	4	0.7205	t test
Appendix Fig S3b	6	0.3419 - 0.0062	One-way ANOVA
Appendix Fig S3c	7	0.0004	One-way ANOVA
Appendix Fig S4c (brain, neutrophils)	4-6	0.0035	One-way ANOVA
Appendix Fig S4c (brain, tumor cells)	4-6	0.0004	One-way ANOVA
Appendix Fig S4c (lungs, neutrophils)	4-6	< 0.0001	One-way ANOVA
Appendix Fig S4c (lungs, tumor cells)	4-6	< 0.0001	One-way ANOVA

Appendix table S3. Statistical data. For each dataset presented in the figures, exact n and p values as well as statistical tests employed are shown.

Appendix figures

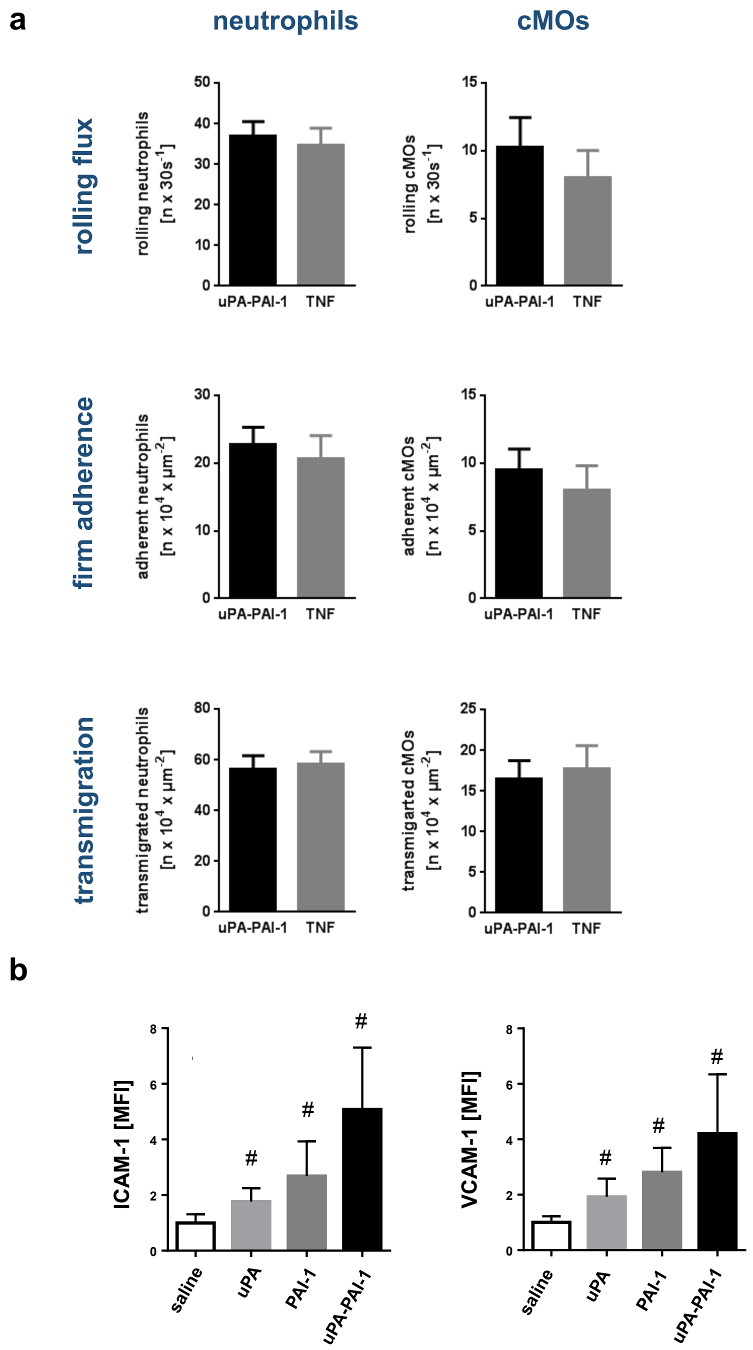

Appendix figure S1. Relative effect of uPA-PAI-1 on the trafficking of neutrophils and cMOs and on endothelial cell activation. (**a**) Intravascular endothelial cell interactions and transmigration of neutrophils (Ly-6G^{+} CX$_3$CR-1^{-}) and cMOs (Ly-6G^{-} CX$_3$CR-1^{low}) to the perivascular tissue as assessed 6 h after intrascrotal stimulation with recombinant murine uPA-PAI-1 or TNF in postcapillary venules of the cremaster of CX$_3$CR-1$^{GFP/+}$ mice by multi-channel *in vivo* microscopy. Quantitative data are shown (mean±SEM for n=4 mice/experiments per group). (**b**) Expression of ICAM-1/CD54 or VCAM-1/CD106 in the cremaster muscle of WT mice as assessed *ex vivo* by confocal laser scanning microscopy 6 h after intrascrotal injection of recombinant murine uPA, PAI-1, uPA-PAI-1, or saline, Quantitative data are shown (mean±SEM for n=4 mice per group; #p<0.05 vs. saline).

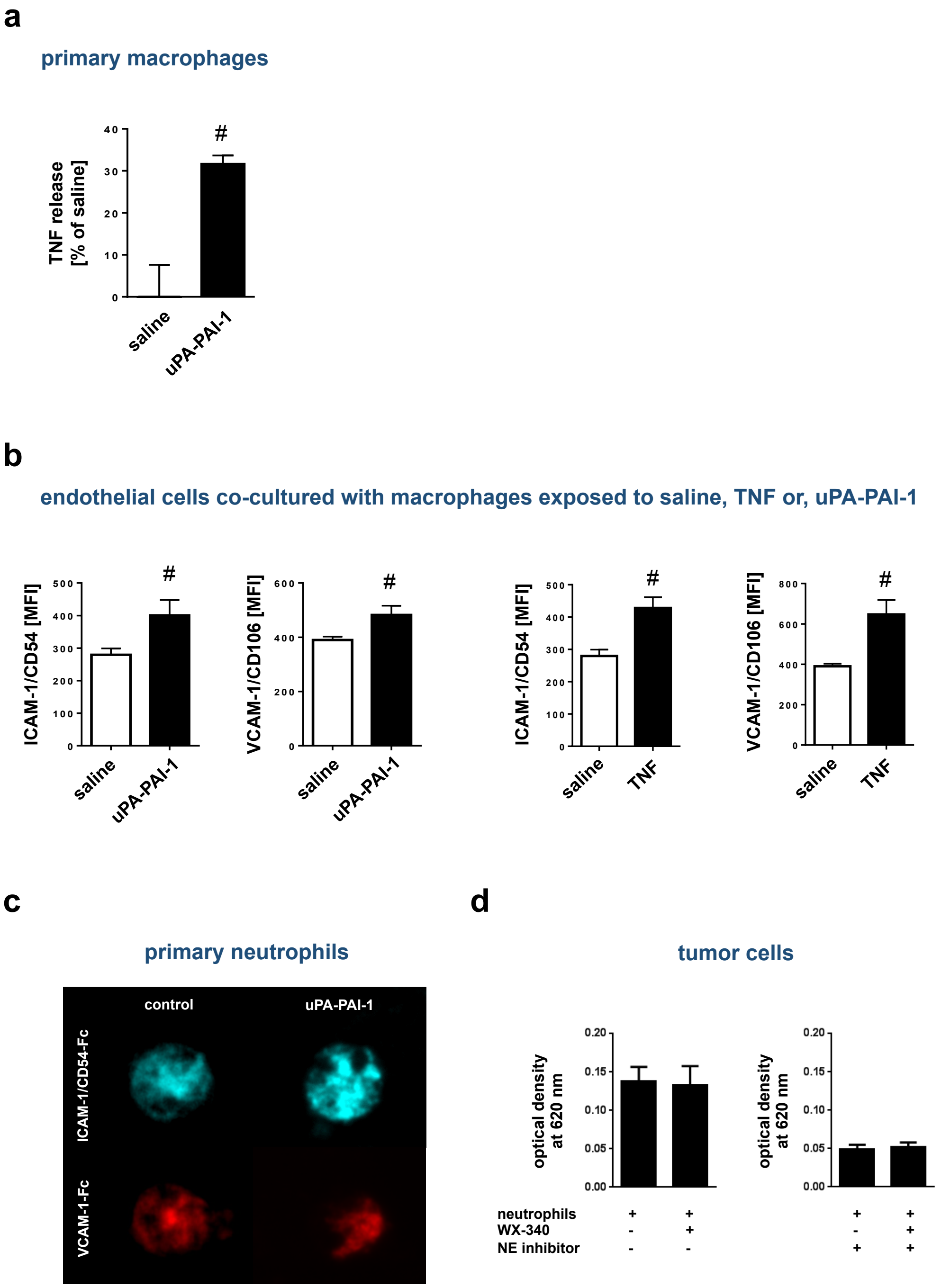

Appendix figure S2. Mechanisms underlying uPA-PAI-1-dependent cell activation. (**a**) Production of TNF was analyzed in peritoneal macrophages harvested from WT mice. (**b**) Surface expression of ICAM-1/CD54 or VCAM-1/CD106 on mouse bEnd.3 microvascular endothelial cells co-cultured with mouse RAW 264.7 macrophages prior to this exposed to saline, recombinant murine uPA-PAI-1, or TNF as assessed *in vitro* by multi-channel flow cytometry. Quantitative data are shown (mean±SEM for n=4-6 experiments per group; #p<0.05 vs. saline). (**c**) Binding of ICAM-1/CD54-Fc or VCAM-1/CD106-Fc to primary mouse neutrophils upon exposure to saline or recombinant murine uPA-PAI-1 as assessed by confocal microscopy, representative images are shown. (**d**) Proliferation of 4T1 breast cancer cells upon exposure to primary neutrophils isolated from the peritoneal cavity of WT mice undergoing 6 h of intra-peritoneal stimulation with uPA-PAI-1 with or without addition of compound WX-340 and a NE inhibitor as assessed by a MTT assay, quantitative data are shown (mean±SEM for n=4 experiments per group).

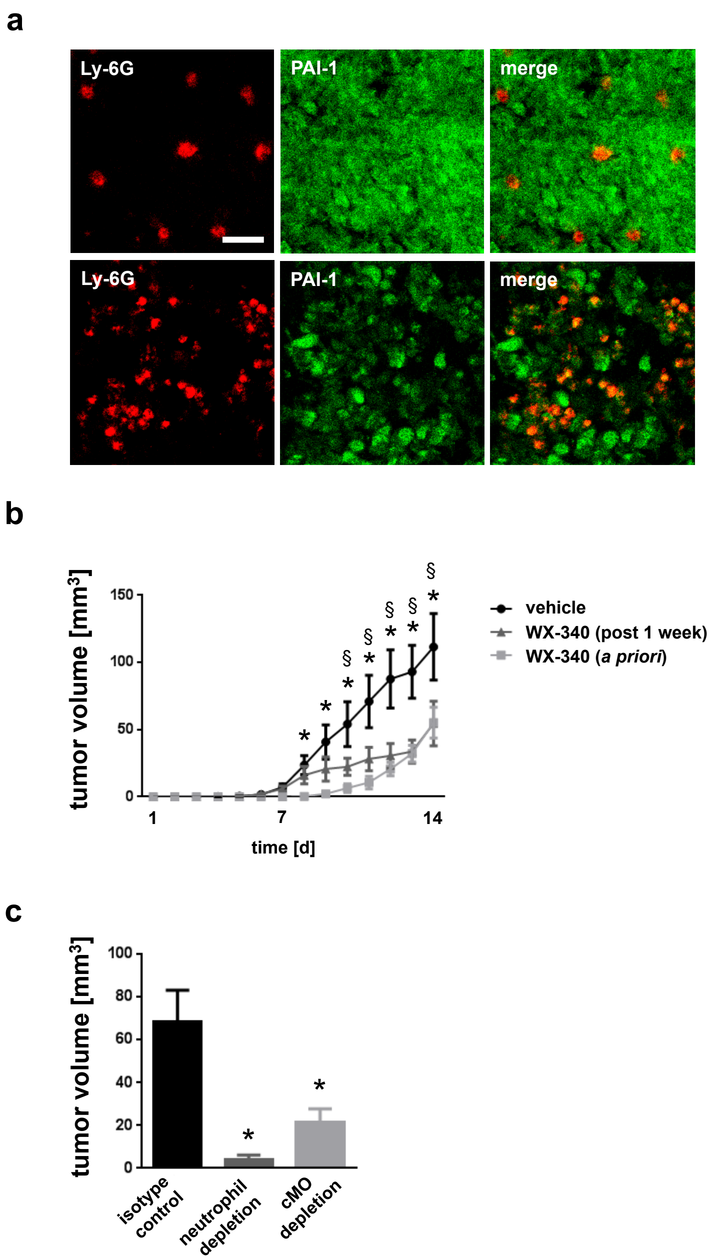

Appendix figure S3. Effect of WX-340 as well as of depletion of neutrophils and cMOs for local tumor growth. (**a**) Representative confocal microscopy images of uPA or PAI-1 expression (green) in mouse 4T1 tumors infiltrated by Ly-6G$^+$ neutrophils (red; scale bar: 25 µm). (**b**) Tumor volume in animals treated with WX-340 *a priori* or therapeutically after 1 week after tumor cell injection on a daily basis as assessed in an orthotopic model of 4T1 breast cancer in WT mice (mean±SEM for n=6 mice per group; *p<0.05 vs. drug vehicle). (**c**) Tumor volume in animals treated with neutrophil- or cMO-depleting monoclonal antibodies as assessed on day 14 after tumor-cell injection in an orthotopic model of 4T1 breast cancer in WT mice (mean±SEM for n=7 mice per group; *p<0.05 vs. isotype control).

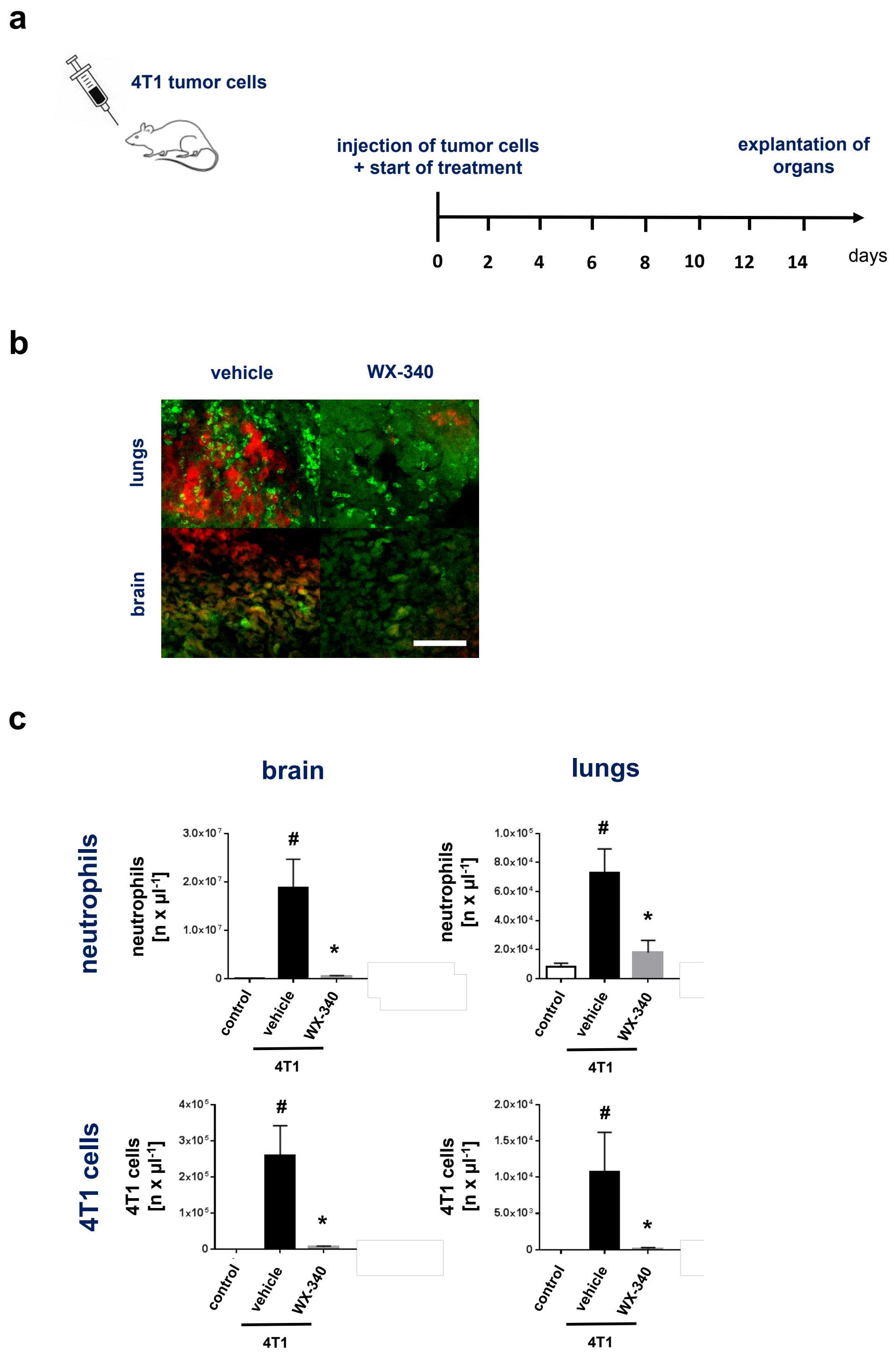

Appendix figure S4. Effect of WX-340 on neutrophil infiltration and metastatic seeding in 4T1 breast cancer metastasis. Accumulation of neutrophils and 4T1 breast cancer cells in lungs and brains of WT mice treated *a priori* intra-peritoneally with WX-340 or drug vehicle (daily) as assessed 14 days after intravenous injection of 4T1 breast cancer cells by multi-channel flow cytometry in the tissue homogenates. (**a**) The experimental protocol, (**b**) representative confocal microscopy images of tumor metastases (red) and neutrophils (green; scale bar: 100 µm) in lungs and brain, and (**c**) quantitative data (mean±SEM for n=4-6 mice per group; #p<0.05 vs. control; *p<0.05 vs. vehicle) are shown.
