# Supplementary material for: uPA‐PAI‐1 heteromerization promotes breast cancer progression by attracting tumorigenic neutrophils
Source: EMBO Mol Med. 2021 May 16;13(6):e13110. doi: 10.15252/emmm.202013110 (PMC8185543; doi:10.15252/emmm.202013110)
Supplement: Supplementary file 2 — Expanded View Figures PDF [file EMMM-13-e13110-s002.pdf]

Expanded View Figures

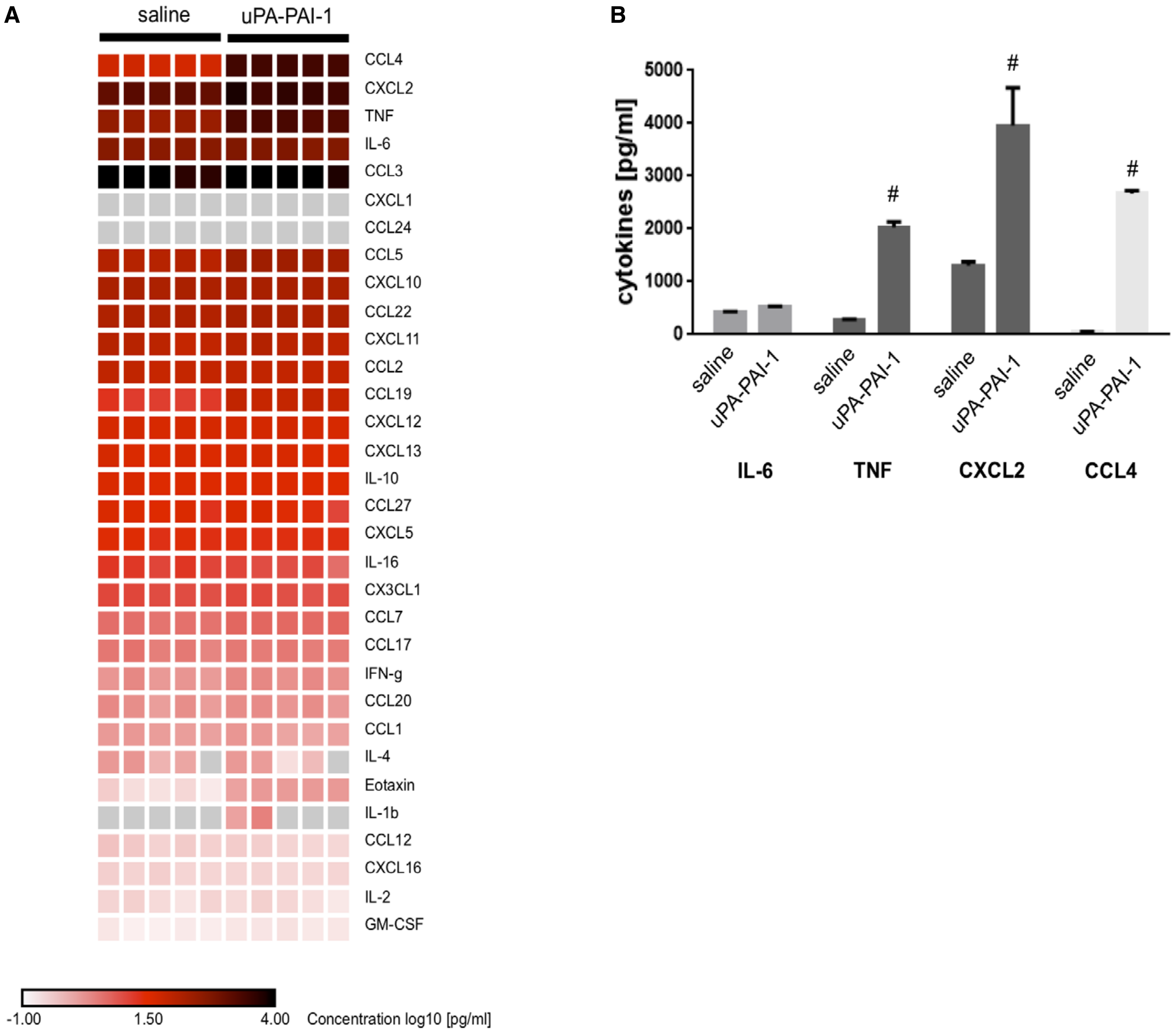

Figure EV1. Effect of uPA-PAI-1 heteromers on cytokine production in macrophages.

A Production of different cytokines by mouse RAW macrophages upon exposure to recombinant murine uPA-PAI-1 heteromers or saline as assessed by multiplex ELISA, results are shown as heatmap.

B In addition, quantitative data are provided for the most abundantly produced cytokines (mean ± SEM for $n = 5$ experiments per group; $^{\#}P < 0.05$ vs. saline; t-test).

© 2021 The Authors

Figure EV2. Molecular mechanisms underlying uPA-PAI-1-dependent neutrophil and cMO trafficking.

A, B uPA-PAI-1-elicited recruitment of neutrophils (N) and classical monocytes (cMOs) to the peritoneal cavity of WT mice treated with anti-LFA-1/CD11a, anti-Mac-1/CD11b, anti-VLA-4/CD49d, anti-ICAM-1/CD54, anti-VCAM-1/CD106 mAbs (A), anti-VLDLr mABs, anti-LRP-1 Abs, the MAPK inhibitors FR180204 (ERK1/2), SB202580 (p38), SP600125 (JNK) (B), or isotype control antibodies/drug vehicle as assessed by multi-channel flow cytometry, quantitative data are shown (mean ± SEM for $n = 4$–6 mice per group; $^{\#}P < 0.05$ vs. saline; $^{*}P < 0.05$ vs. isotype/vehicle; one-way ANOVA).

C Recruitment of neutrophils (N) and classical monocytes (cMOs) to the peritoneal cavity of WT mice treated with recombinant murine uPA-PAI-1, recombinant human uPA-recombinant murine PAI-1 heteromers, recombinant murine DFP-uPA-PAI-1, or vehicle (mean ± SEM for $n = 4$–6 mice per group; $^{\#}P < 0.05$ vs. saline; one-way ANOVA).

© 2021 The Authors

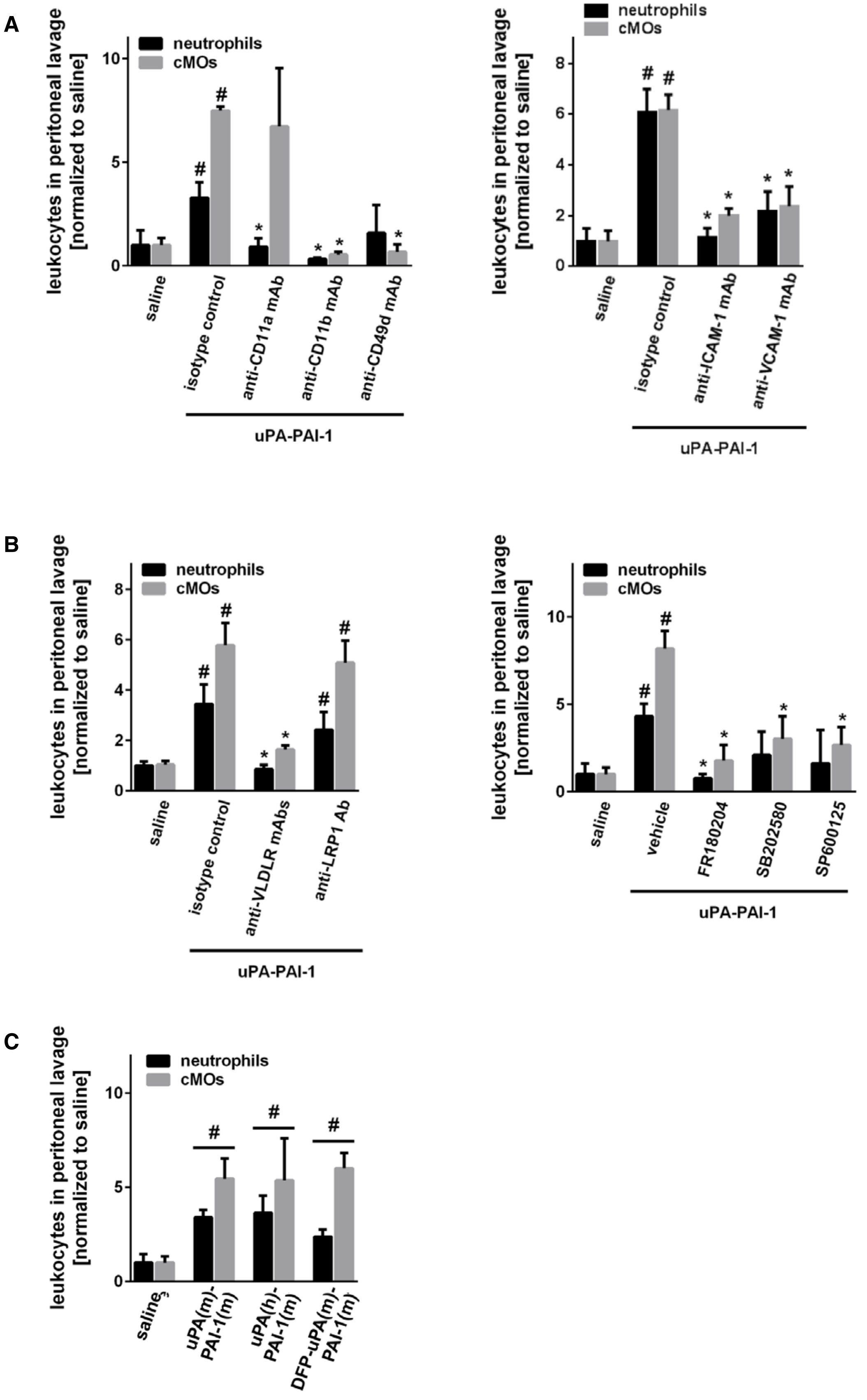
A
B
C
leukocytes in peritoneal lavage [normalized to saline]
neutrophils
cMOs
saline
isotype control
anti-CD11a mAb
anti-CD11b mAb
anti-CD49d mAb
anti-ICAM-1 mAb
anti-VCAM-1 mAb
anti-VLDLR mAbs
anti-LRP1 Ab
vehicle
FR180204
SB202580
SP600125
uPA-PAI-1
uPA(m)-PAI-1(m)
uPA(h)-PAI-1(m)
DFP-uPA(m)-PAI-1(m)

Figure EV2.

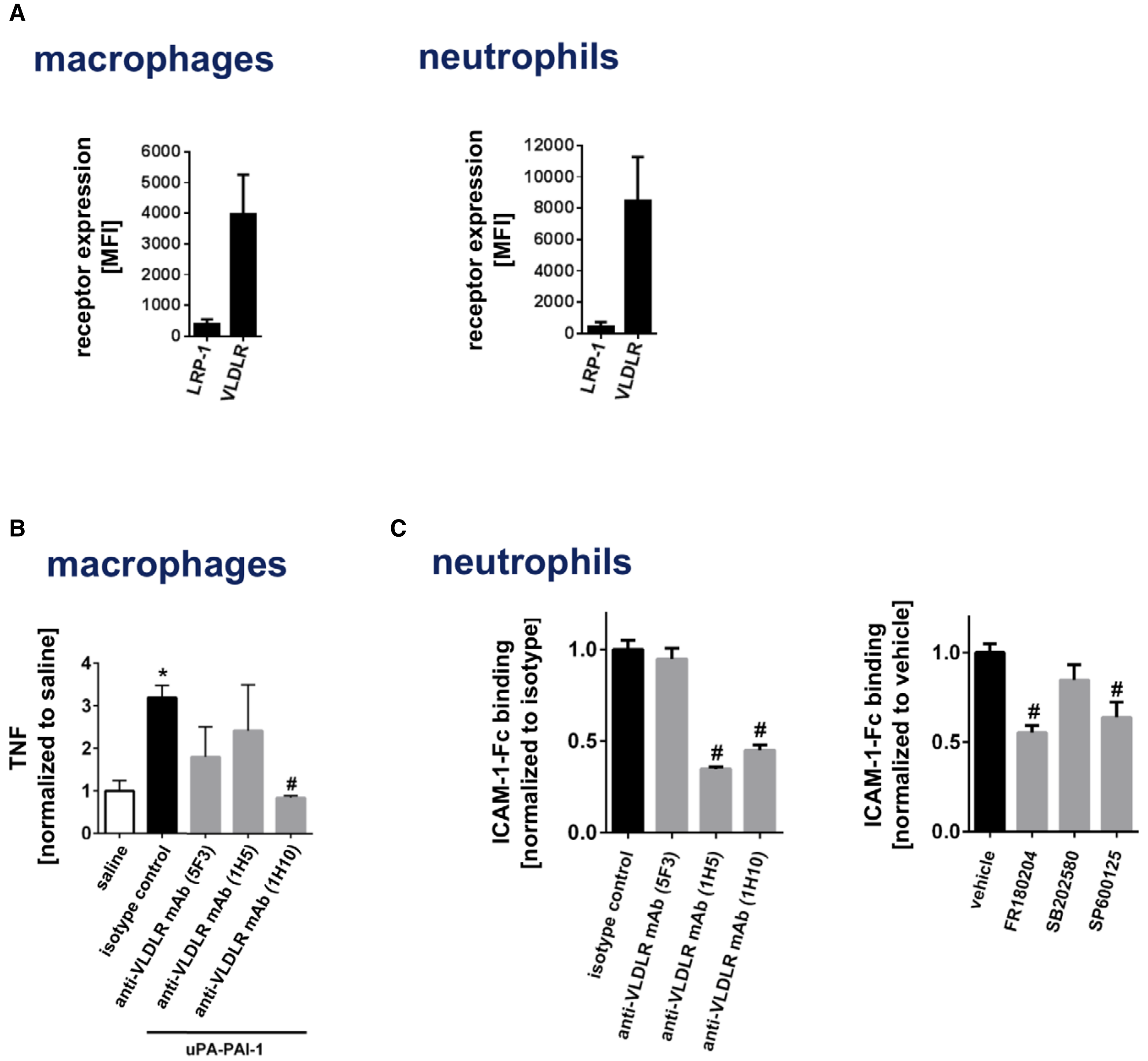

Figure EV3. Molecular mechanisms underlying uPA-PAI-1-dependent responses of macrophages and neutrophils.

A Surface expression of LRP-1 or VLDLr on mouse RAW macrophages or bEnd.3 endothelial cells (mean ± SEM for n = 3 experiments per group).

B Production of TNF by mouse RAW macrophages upon exposure to blocking antibodies directed against different epitopes of VLDLr as assessed by multi-channel flow cytometry (mean ± SEM for n = 4–6 experiments per group; $^{*}P < 0.05$ vs. saline; $^{\#}P < 0.05$ vs. isotype control; one-way ANOVA).

C Binding of recombinant murine ICAM-1/CD54-Fc to blood neutrophils from WT mice upon exposure to blocking antibodies directed against different epitopes of VLDLr or the MAPK inhibitors FR180204 (ERK), SB202580 (p38), or SP600125 (JNK) as assessed by flow cytometry (mean ± SEM for n = 4–6 mice per group; $^{\#}P < 0.05$ vs. isotype control/vehicle; one-way ANOVA).

Figure EV4. Correlation of neutrophil infiltration and uPA/PAI-1 expression in human breast cancer samples. ▶

A, B Correlation of uPA or PAI-1 protein expression (ELISA) and neutrophil infiltration (histochemistry and light microscopy) in human breast cancer samples, representative images (A; histological grades: G1-3; scale bar: 100 µm) and quantitative data (B; histological grades: G2 or G3; n = 10–29 samples per group).

 © 2021 The Authors

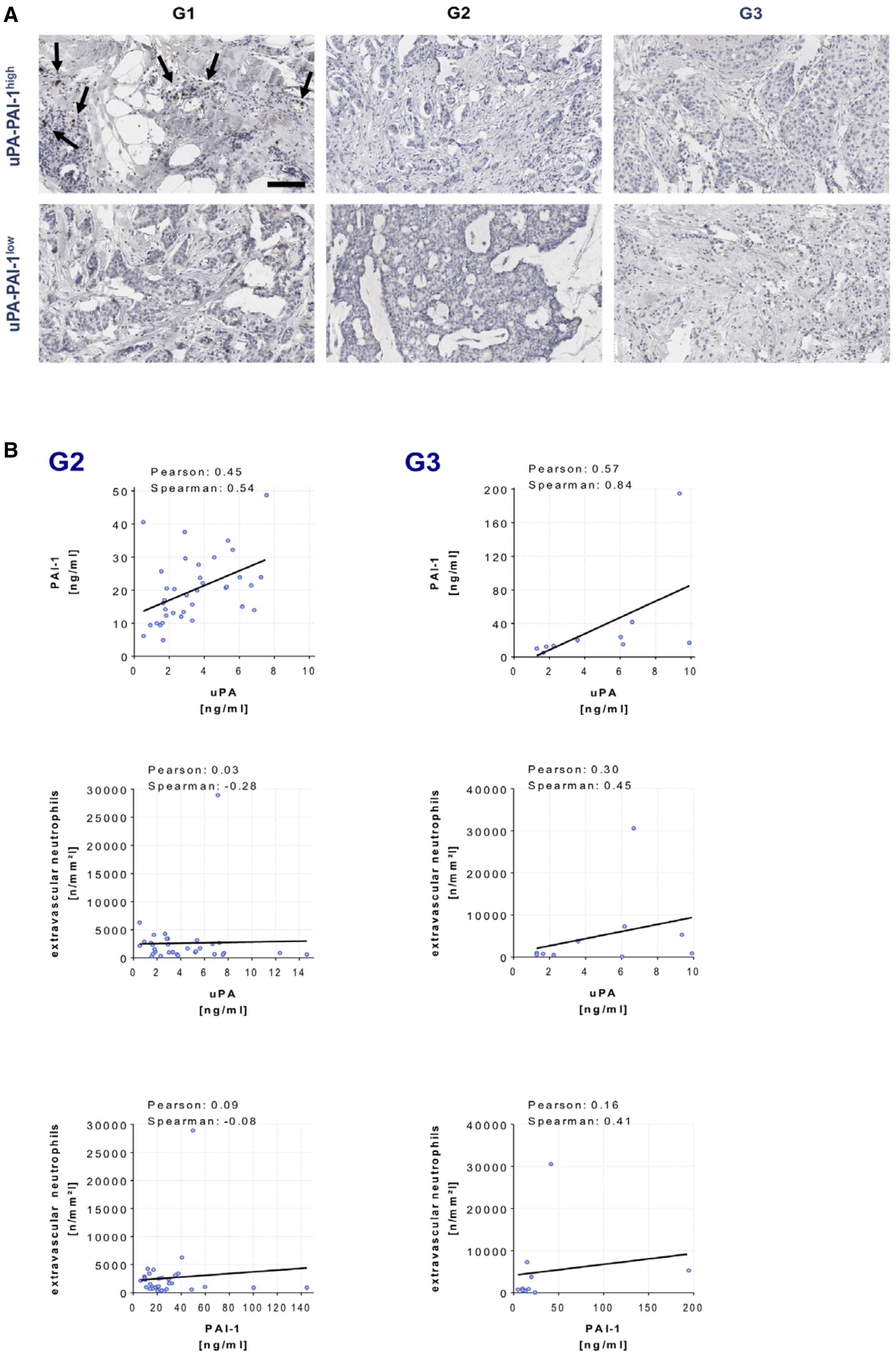
A
G1
G2
G3
uPA-PAI-1high
uPA-PAI-1low
B
G2
G3
Pearson: 0.45
Spearman: 0.54
Pearson: 0.57
Spearman: 0.84
PAI-1 [ng/ml]
uPA [ng/ml]
Pearson: 0.03
Spearman: -0.28
Pearson: 0.30
Spearman: 0.45
extravascular neutrophils [n/mm²]
Pearson: 0.09
Spearman: -0.08
Pearson: 0.16
Spearman: 0.41
PAI-1 [ng/ml]

Figure EV4.

Figure EV5. Overall survival of breast cancer patients of the METABRIC cohort with respect to disease stage.

A Composition of analyzed patients of the METABRIC cohort with respect to the disease stage.

B Overall survival of PLAU-SERPINE1^{low} and PLAU-SERPINE1^{high} breast cancer patients in all disease stages (0–4) and advanced stages (2–4).

C Mosaic plot depicting cross-tabulation and chi-squared analysis between high RNA expression of PLAU or SERPINE1 in the tumor and the molecular breast cancer subtype as defined by the 3-gene-classifier.

© 2021 The Authors

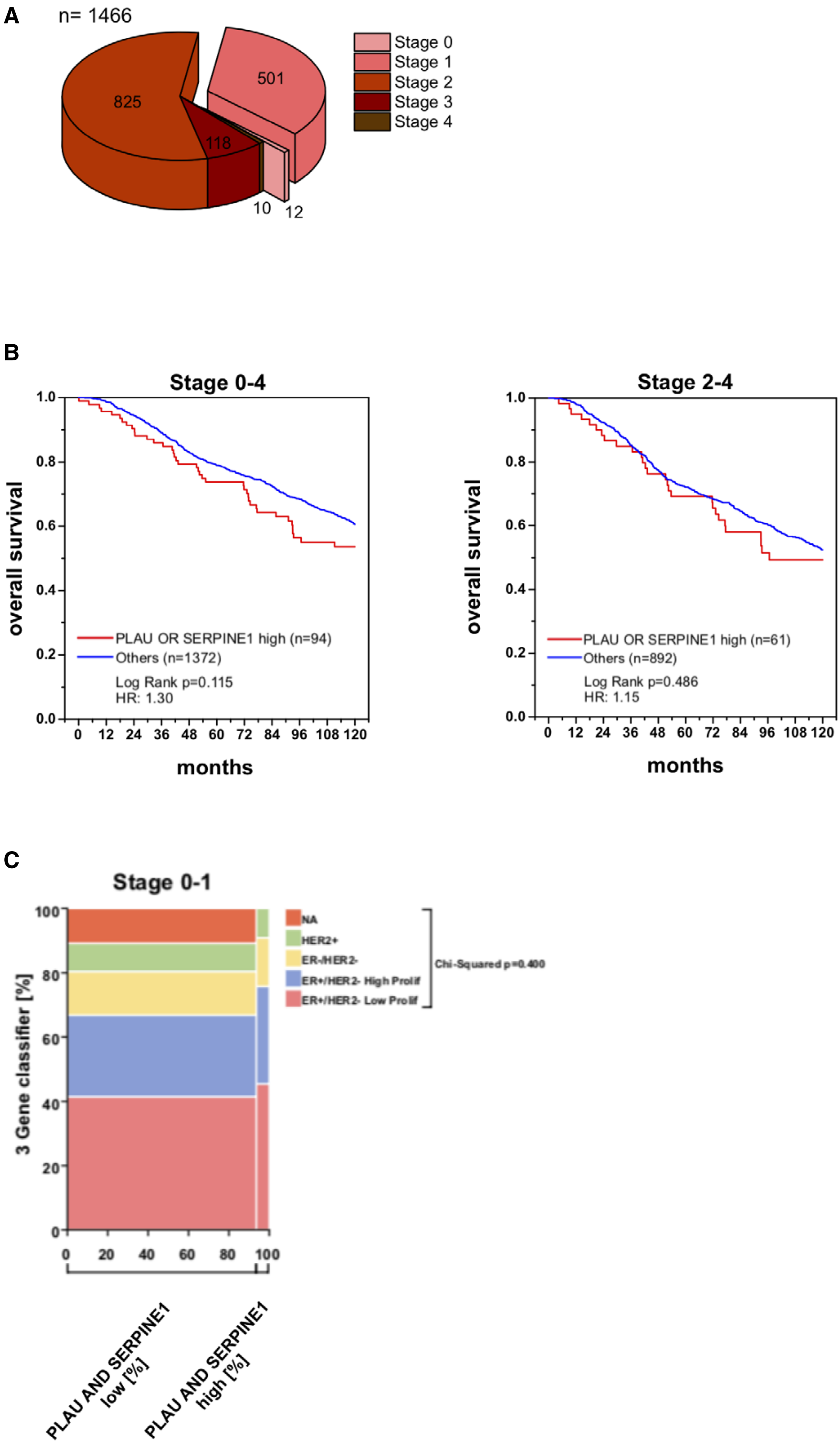
A
n= 1466
825
501
118
10
12
Stage 0
Stage 1
Stage 2
Stage 3
Stage 4
B
Stage 0-4
overall survival
months
PLAU OR SERPINE1 high (n=94)
Others (n=1372)
Log Rank p=0.115
HR: 1.30
Stage 2-4
PLAU OR SERPINE1 high (n=61)
Others (n=892)
Log Rank p=0.486
HR: 1.15
C
Stage 0-1
3 Gene classifier [%]
NA
HER2+
ER-/HER2-
ER+/HER2- High Prolif
ER+/HER2- Low Prolif
Chi-Squared p=0.400
PLAU AND SERPINE1 low [%]
PLAU AND SERPINE1 high [%]

Figure EV5.
